# Characteristics, barriers, and career intentions of a national cohort of LGBTQ+ MD/PhD and DO/PhD trainees

**DOI:** 10.1186/s12909-022-03378-8

**Published:** 2022-04-21

**Authors:** Mollie C. Marr, Anna S. Heffron, Jennifer M. Kwan

**Affiliations:** 1 Oregon Health & Science University Medical Scientist Training Program, Portland, OR USA; 2 University of Wisconsin School of Medicine and Public Health Medical Scientist Training Program, Madison, WI USA; 3 Yale School of Medicine in New Haven, New Haven, CT 06520 USA

**Keywords:** LGBTQ+, Physician-scientist, MD/PhD, DO/PhD, Medical education

## Abstract

**Background:**

Lesbian, gay, bisexual, transgender, queer, non-binary, intersex, and/or asexual (LGBTQ+) individuals continue to suffer worse health outcomes compared to the general population. Data on LGBTQ+ individuals in medicine, particularly in medical training, remain sparse. National studies of LGBTQ+ students in MD/PhD and DO/PhD training programs have not been reported.

**Methods:**

Trainees pursuing MD, DO, MD/PhD, and DO/PhD degrees at 32 nationally representative institutions completed a 70-item survey about their future career and anticipated challenges using an online survey tool from September 2012 to December 2014. There were 4,433 respondents to the survey. Of those, 2,837 completed the gender identity questions and 2,849 completed the sexual orientation questions. Completion of these questions was required for inclusion. Survey results were analyzed to examine differences between LGBTQ+ and non-LGBTQ+ medical and dual degree trainees.

**Results:**

LGBTQ+ students were underrepresented among MD/PhD and DO/PhD trainees (8.70%) compared to the US population, though their representation was higher than among MD and DO trainees (5.20%). LGBTQ+ dual degree trainees endorsed the greatest interest in pursuing careers involving academic medicine, with varying career focuses including research, clinical duties, education, and advocacy. LGBTQ+ dual degree trainees prioritized opportunities in patient care, work-life balance, and research as the most important factors for their career selection. Importantly, a higher percentage of LGBTQ+ dual degree trainees (15.50%) identified sexual harassment as a past barrier to career advancement compared to their non-LGBTQ+ peers (8.27%). LGBTQ+ dual degree trainees were more likely to report having a mentor who advocated for them.

**Conclusions:**

LGBTQ+ physician scientist trainees remain under-represented and under-studied. It is vital that medical institutions devote more time and resources towards identifying and addressing the unique needs of this group in training. Training programs should be aware of the current and prior challenges faced by their LGBTQ+ dual degree trainees, work to overcome the unique barriers they face, highlight the strengths and unique perspectives they bring, and foster their professional growth and goals during and beyond their training.

**Supplementary Information:**

The online version contains supplementary material available at 10.1186/s12909-022-03378-8.

## Background

Individuals identifying as lesbian, gay, bisexual, transgender, queer, intersex non-binary and/or non-cisgender (LGBTQ+) make invaluable contributions to medicine and science. LGBTQ+ patients report feeling more comfortable accessing medical care when their provider is also LGBTQ+ or is familiar with the LGTBQ+ community [1–3], which is particularly important given the wide health disparities for LGBTQ+ individuals [4]. Accurate scientific work concerning the LGBTQ+ community benefits enormously from the presence and knowledge of LGBTQ + researchers [5], and LGBTQ+ scientists have been responsible for pivotal scientific advances, particularly for the health of the LGBTQ+ community. LGBTQ+ trainees in science and medicine benefit from LGBTQ+ role models and mentorship [6, 7]. All trainees benefit from exposure to LGBTQ+ leaders and leaders familiar with the LGBTQ+ community [8]. Thus, it is vital to ensure that LGBTQ+ people are comfortable, confident, and supported in their training as physicians, researchers, and physician-scientists.

### LGBTQ+ in STEM

Despite the importance of LGBTQ+ representation, LGBTQ+ people are highly underrepresented in medicine and science. LGBTQ+ people are estimated to comprise 10–15% of the US population [9], with increasing representation in younger generations, but the American Medical Association estimates that only about 4% of US physicians identify as LGBTQ+ [10]. LGBTQ+ representation among physician-researchers has not been reported. Recent studies have shown that LGBTQ+ undergraduate students are 7% less likely to be retained in science, technology, engineering and mathematics (STEM) fields [11]. LGBTQ+ medical students are more likely to experience burnout, which is associated with their higher likelihood of mistreatment [12]. Though national data on LGBTQ+ PhD students have not been examined [13], a study at one institution found these students are more likely to not complete their programs [14]. Higher rates of attrition among LGBTQ+ undergraduate, [15] middle, and high school students have been established [16]. LGBTQ+ physicians commonly face discriminatory behavior from patients, with 42% of residents in a recent survey reporting witnessing discrimination within the past year [17]. A study of physical scientists in the United Kingdom found that 28% of LGBTQ+ scientists and nearly 50% of all transgender scientists have considered leaving their workplace due to a hostile climate or discrimination [18]. Status as an LGBTQ+ person in STEM professional fields is an independent predictor of experiencing career limitations, harassment, professional devaluation, more frequent health difficulties, and greater likelihood of intending to leave STEM [19]. LGBTQ+ trainees face further challenges as they are not recognized as an underrepresented group [20–22]. Heteronormative assumptions and environments often silence conversations about gender and sexuality in medicine and STEM workplaces, leading LGBTQ+ trainees and scientists to report feeling “invisible” [5, 23–29]. Concerns of LGBTQ+ trainees in combined dual degree programs, such as MD/PhD and DO/PhD, are truly invisible, since data on this subgroup has not previously been collected or analyzed on a national level. Considering the instrumental role that physician-scientists play in pivotal advances in health science, the paucity of data on tools for success and barriers in training for this group is concerning.

### Present study

The objective of the present study was to examine the characteristics of LGBTQ+ MD/PhD and DO/PhD trainees, factors associated with career interests and goals of this group, and potential barriers experienced by LGBTQ+ physician-scientists amongst a national cohort of trainees. We hypothesized that LGBTQ+ dual degree trainees represent a unique cohort with distinct experiences and barriers related to their academic and research careers. Determining these unique characteristics represents an important step to ensuring LGBTQ+ trainees have access to a more equitable training environment allowing them to reach their full potential as future physician-scientists.

## Methods

### Study design

The study methods have been previously published [30, 31]. Briefly, medical trainees at 32 nationally representative institutions completed a 70-item survey about their future careers and anticipated challenges using an online survey tool from September 2012 to December 2014 (for full survey, see supplemental materials). The survey was sent to all medical trainees at these institutions. Recruitment was through email listservs distributed by institutional representatives of the American Physician Scientist Association. The study was reviewed and exempted by the Institutional Review Boards at the University of Illinois at Chicago and University of Pennsylvania.

In the present study, “inmedical trainees” refers to both MD and DO trainees. “Dual degrees trainees” refers to both MD/PhD and DO/PhD trainees. LGBTQ+ was defined as those who indicated that they were transgender female, transgender male, queer, genderqueer, gay, lesbian or bisexual on the sexual orientation and/or gender identity questions.

### Statistical analysis

Survey results were analyzed to examine differences between LGBTQ+ and non-LGBTQ+ medical and dual degree trainees in career interests, barriers, and support. Descriptive statistics were employed due to low numbers in the LGBTQ+ groups. All analyses were performed using SPSS version 24 [32].

## Results

### Respondent characteristics

There were 4,433 respondents to the survey, representing a 27% response rate. Of those respondents, 2,837 completed the question about gender identity and 2,849 answered the question about sexual orientation. More men, regardless of LGBTQ+ identity, were enrolled in dual degree programs compared to women. The difference was most pronounced for LGBTQ+ trainees, where 56.52% of dual degree trainees were male and 41.30% were female. There was only one dual degree transgender woman and no transgender men, and one queer/genderqueer dual degree student. Among medical trainees, there were four transgender women and no transgender men. The majority of all trainees were white and non-Latinx. More MD- or DO-only first year students responded (32%) compared to other years of medical training. Dual degree trainees in medical training responded in similar numbers regardless of training year (Range: 8.8—13.3%). Responses of dual degree trainees during the first 4 years of graduate school, which most students complete, ranged from 4.44% to 22.22%. Demographic characteristics of survey respondents segregated by LGBTQ+ or non-LGBTQ+ are summarized in Table 1.

**Table 1:** Respondent characteristics by LGBTQ+ and dual degree and medical trainees (*n* = 2,841)

	**LGBTQ+ **		**non-LGBTQ+ **	
	**MD/PhD or DO/PhD n (%)**	**MD/DO n (%)**	**MD/PhD or DO/PhD n (%)**	**MD/DO n (%)**
**Sex**				
Male	26 (56.52)	63 (52.50)	264 (54.65)	883 (40.35)
Female	18 (39.13)	49 (40.83)	219 (45.34)	1305 (59.64)
Transgender female^a^	1 (2.17)	4 (3.33)	0 (0)	0 (0)
Queer/Genderqueer	1 (2.17)	4 (3.33)	0 (0)	0 (0)
**Total (n)**	**46**	**120**	**483**	**2188**
**Race**				
White	33 (71.73)	76 (66.08)	329 (68.54)	1479 (68.25)
Black or African American	2 (4.34)	2 (1.73)	20 (4.16)	98 (4.52)
American Indian or Alaska Native	0 (0)	0 (0)	1 (0.20)	6 (0.27)
Asian or Pacific Islander	8 (17.39)	19 (16.52)	76 (15.83)	370 (17.07)
Multiracial or Not listed above	3 (6.52)	18 (15.65)	54 (11.25)	214 (9.87)
**Total (n)**	**46**	**115**	**480**	**2167**
**Ethnicity**				
Hispanic/Latinx/Latine	4 (8.69)	10 (8.62)	26 (5.42)	140 (6.43)
Not Hispanic/Latinx/Latine	42 (91.30)	106 (91.37)	453 (94.57)	2037 (93.56)
**Total (n)**	**46**	**116**	**479**	**2177**
**Year**				
Medical School Year 1	6 (13.33)	37 (31.89)	97 (20.04)	707 (32.19)
Medical School Year 2	5 (11.11)	40 (34.48)	77 (15.90)	573 (26.09)
Medical School Year 3	4 (8.88)	22 (18.96)	36 (7.44)	423 (19.26)
Medical School Year 4	6 (13.33)	13 (11.20)	43 (8.88)	387 (17.62)
Graduate School Year 1	10 (22.22)	0 (0)	66 (13.63)	19 (0.87)
Graduate School Year 2	2 (4.44)	0 (0)	54 (11.15)	6 (0.27)
Graduate School Year 3	5 (11.11)	0 (0)	37 (7.64)	5 (0.23)
Graduate School Year 4	6 (13.33)	0 (0)	47 (9.71)	5 (0.23)
Graduate School Year 5	0 (0)	0 (0)	9 (1.86)	2 (0.09)
Graduate School Year 6 +	0 (0)	0 (0)	7 (1.45)	1 (0.05)
Year out for Research	0 (0)	4 (3.45)	6 (1.24)	43 (1.96)
Other or N/A^b^	1 (2.22)	0 (0)	9 (1.86)	25 (1.14)
**Total (n)**	**45**	**116**	**484**	**2,196**

^a^No trainees identified as transgender male

^b^Individuals who thought they did not fit into predefined selection options chose “Other” or “N/A.” Some indicated they were taking a break, some that they were doing more extensive research programs, and some did not give a reason

### LGBTQ+ trainees endorse greatest interest in academic careers, education, and advocacy

The majority of trainees, regardless of LGBTQ+ identity, indicated a plan to continue onto residency training (Table 2). The predominant intended career of all dual degree trainees was academia (LGBTQ+ : 88.88%, non-LGBTQ+ : 83.11%). LGBTQ+ medical trainees also expressed slightly greater interest in academic medicine compared to non-LGBTQ+ medical trainees (41.28% vs. 35.79%). All LGBTQ+ trainees expressed slightly less interest in private practice compared to non-LGBTQ+ trainees (Dual degree: 0% vs. 4.97%; Medical: 23.85% vs. 32.31%).

**Table 2:** Career sector, focus, and priorities of LGBTQ+ and dual degree and medical trainees (*n* = 2,754)

	**LGBTQ+ **			**non-LGBTQ+ **
	**MD/PhD or DO/PhD n (%)**	**MD/DO n (%)**	**MD/PhD or DO/PhD n (%)**	**MD/DO n (%)**
**Plan to go onto residency training**				
Yes	44 (97.77)	113 (99.12)	461 (97.66)	2093 (98.58)
No	1 (2.22)	1 (0.87)	11 (2.33)	30 (1.41)
**Total (n)**	**45**	**114**	**472**	**m**
**Career sector**				
Consulting	2 (4.44)	2 (1.83)	9 (1.94)	41 (1.98)
Academia	40 (88.88)	45 (41.28)	384 (83.11)	741 (35.79)
Industry	0 (0)	0 (0)	9 (1.94)	29 (1.40)
Government	1 (2.22)	3 (2.75)	8 (1.73)	64 (3.09)
Private Practice	0 (0)	26 (23.85)	23 (4.97)	669 (32.31)
Hospitalist	2 (4.44)	26 (23.85)	25 (5.41)	462 (22.3)
Other	0 (0)	6 (5.50)	3 (0.64)	51 (2.46)
N/A	0 (0)	1 (0.91)	1 (0.21)	13 (0.62)
**Total (n)**	**45**	**109**	**462**	**2070**
**Career focus**				
Education	3 (6.66)	10 (9.17)	6 (1.29)	145 (7.00)
Basic research	8 (17.77)	0 (0)	101 (21.8)	15 (0.72)
Clinical research	3 (6.66)	6 (5.50)	29 (6.27)	106 (5.12)
Translational research	17 (37.77)	5 (4.58)	198 (42.8)	62 (2.99)
Clinical duties	8 (17.77)	78 (71.55)	99 (21.42)	1586 (76.61)
Therapeutics/diagnostics	2 (4.44)	0 (0)	14 (3.03)	24 (1.15)
Advocacy	1 (2.22)	6 (5.50)	2 (0.43)	53 (2.56)
Administration	0 (0)	0 (0)	0 (0)	44 (2.12)
NA	0 (0)	1 (0.91)	0 (0)	13 (0.62)
Other	3 (6.66)	3 (2.75)	11 (2.38)	17 (0.82)
**Total (n)**	**45**	**109**	**460**	**2065**
**Most important factors in career selection**				
Opportunities to do research	9 (20.00)	3 (2.77)	187 (40.30)	69 (3.356)
Opportunities for patient care	12 (26.66)	40 (37.03)	75 (16.16)	789 (38.37)
Opportunities to teach	2 (4.44)	9 (8.33)	10 (2.15)	42 (2.04)
Opportunities for community service	1 (2.22)	4 (3.70)	4 (0.86)	81 (3.93)
Opportunities for interactions with students	0 (0)	0 (0)	0 (0)	25 (1.21)
Opportunities for travel	0 (0)	2 (1.85)	1 (0.21)	13 (0.63)
Opportunities for international work	2 (4.44)	3 (2.77)	5 (1.07)	58 (2.82)
Opportunities for national work	0 (0)	0 (0)	3 (0.64)	10 (0.48)
Opportunities for local work	0 (0)	1 (0.92)	2 (0.43)	10 (0.48)
Ability to balance work and personal life	10 (22.22)	33 (30.55)	135 (29.09)	792 (38.52)
Financial security	3 (6.66)	9 (8.33)	18 (3.87)	89 (4.32)
Autonomy	4 (8.88)	3 (2.77)	17 (3.66)	39 (1.89)
Prestige	0 (0)	0 (0)	0 (0)	7 (0.34)
Other	2 (4.44)	1 (0.92)	7 (1.50)	32 (1.55)
**Total (n)**	**45**	**108**	**464**	**2056**

The top three areas of career focus for all dual degree trainees were translational research (37.77%), basic research (17.77%), and clinical duties (17.77%) (Table 2). Dual degree LGBTQ+ trainees also expressed greater interest in education (6.66% vs.1.29%) and advocacy (2.22% vs. 0.43%) compared to non-LGBTQ+ dual degree trainees. Although we were not able to statistically compare the write-in responses between LGBTQ+ trainees and non-LGBTQ+ trainees because of the low number of LGBTQ+ trainees, public health and policy work were mentioned as areas of career focus by several LGBTQ+ trainees.

### LGBTQ+ trainees prioritize opportunities for patient care, work-life balance, and research in career selection

LGBTQ+ trainees identified opportunities for patient care (Dual degree: 26.66%; Medical: 37.03%) and ability to balance work and personal life (Dual degree: 22.22%; Medical: 30.55%) as the most important factors in career selection (Table 2). LGBTQ+ dual degree trainees also endorsed opportunities to do research (20.00%) as one of the most important factors in career selection. Non-LGBTQ+ dual degree trainees identified the same key factors, but in a different order of importance. A greater number of non-LGBTQ+ dual degree trainees selected opportunities to do research as the most important factor in career selection compared to LGBTQ+ dual degree trainees (40.30% vs. 20.00%). Among non-LGBTQ+ trainees, ability to balance work and personal life (Dual degree: 29.09%; Medical: 38.52%) and opportunities for patient care (Dual degree: 16.16%; Medical: 38.37%) were among the top factors endorsed. Reflecting career intentions, a greater number of dual degree and medical LGBTQ+ trainees selected opportunities to teach compared to non-LGBTQ+ trainees (Dual degree: 4.44% vs. 2.15%; Medical: 8.33% vs. 2.04%).

### LGBTQ+ dual degree trainees identify shared and unique career hindrances compared to their non-LGBTQ+ peers

Balancing family and work responsibilities was identified as the most pressing career obstacle by all trainees (Table 3). The second and third most endorsed barriers, lack of opportunity/funding and balancing clinical, research, and education, were the same for all dual degree trainees. LGBTQ+ dual degree trainees also identified not finding a position in a desired location as a top barrier.

**Table 3:** Past and potential barriers to career advancement (*n* = 2,663). Respondents first selected the single most pressing career obstacle. They next selected all hindrances that they have encountered in their career; therefore, for this question, percentages were calculated based on the total number of survey respondents in each category

	**LGBTQ+ **		**non-LGBTQ+ **	
	**MD/PhD or DO/PhD n (%)**	**MD/DO n (%)**	**MD/PhD or DO/PhD n (%)**	**MD/DO n (%)**
**Most pressing career obstacle**				
Lack of opportunity/funding	11 (24.44)	2 (1.83)	125 (27.23)	78 (3.80)
Not finding position in desired location	5 (11.11)	12 (11.00)	34 (7.40)	190 (9.26)
Loan repayment	1 (2.22)	19 (17.43)	10 (2.17)	347 (16.92)
Malpractice/lawsuit	0 (0)	1 (0.91)	1 (0.21)	42 (2.04)
Under-compensation	1 (2.22)	2 (1.83)	5 (1.08)	97 (4.73)
Discrimination/biases against your gender/ethnicity/sexual orientation	3 (6.66)	8 (7.33)	3 (0.65)	19 (0.92)
Sexual harassment	1 (2.22)	0 (0)	0 (0)	1 (0.04)
Balancing family and work responsibilities	12 (26.66)	51 (46.78)	173 (37.69)	1082 (52.78)
Balancing clinical, research, and education responsibilities	5 (11.11)	7 (6.422)	92 (20.04)	124 (6.04)
Satisfactory professional advancement	3 (6.66)	6 (5.50)	13 (2.83)	50 (2.43)
Other	3 (6.66)	1 (0.91)	3 (0.65)	20 (0.97)
**Total (n)**	**45**	**109**	**459**	**2050**
**Past hindrances to career advancement**				
Lack of opportunity/funding	12 (26.66)	18 (16.50)	91 (19.82)	389 (18.90)
Not finding position in desired location	4 (8.88)	9 (8.26)	44 (9.58)	154 (7.51)
Loan repayment	1 (2.22)	24 (22.01)	33 (7.18)	482 (23.51)
Malpractice/lawsuit	0 (0)	1 (0.91)	1 (0.21)	19 (0.92)
Under-compensation	2 (4.44)	6 (5.50)	43 (9.36)	123 (6.00)
Discrimination/biases against your gender/ethnicity/sexual orientation	3 (6.66)	8 (7.33)	3 (0.65)	19 (0.92)
Sexual harassment	7 (15.50)	17 (15.50)	38 (8.27)	134 (6.53)
Balancing family and work responsibilities	2 (4.44)	4 (3.67)	6 (1.31)	18 (0.88)
Balancing clinical, research, and education responsibilities	20 (44.44)	52 (47.70)	167 (36.38)	835 (40.70)
Satisfactory professional advancement	4 (8.88)	15 (13.70)	59 (12.80)	190 (9.26)
Other	5 (11.10)	3 (2.75)	17 (3.70)	76 (3.70)
**Total (n)**	**45**	**109**	**459**	**2050**

Respondents also identified past hindrances to career advancement (Table 3). All trainees endorsed past barriers related to balancing clinical, research, and educational opportunities. Over 25% of LGBTQ+ and 19.82% of non-LGBTQ+ dual degree trainees endorsed a lack of opportunity/funding as a past barrier. The next most highly endorsed hindrance by LGBTQ+ dual degree trainees was sexual harassment (15.50%) while non-LGBTQ+ dual degree trainees next endorsed concerns about satisfactory professional advancement (12.80%).

### Effective mentoring is vital to all trainees

A greater percentage of dual degree trainees indicated that they could identify a mentor who has helped with their progress toward career goals compared to all medical trainees. Similarly, more dual degree trainees, compared to medical trainees, said that their mentor had advocated for them beyond feedback or advice and had used their influence to advocate for awards, fellowships, and promotions. Importantly, a majority of LGBTQ+ dual degree trainees (63.63%) reported that their mentors advocated for them compared to non-LGBTQ+ dual degree trainees (44.69%). Nearly all of the LGBTQ+ dual degree (97.62%) and a majority of the non-LGBTQ+ dual degree (93.30%) trainees described mentorship as being somewhat or very important to their careers (Table 4).

**Table 4:** Role of mentorship for LGBTQ+ and dual degree and medical trainees (*n* = 2,637)

	**LGBTQ+ **		**non-LGBTQ+ **	
	**MD/PhD or DO/PhD n (%)**	**MD/DO n (%)**	**MD/PhD or DO/PhD n (%)**	**MD/DO n (%)**
**Can identify a mentor**				
Yes	42 (93.33)	76 (70.37)	424 (92.37)	1452 (71.70)
No	3 (6.66)	32 (29.62)	35 (7.62)	573 (28.29)
**Total (n)**	**45**	**108**	**459**	**2025**
**Mentor advocates for you**				
Yes	28 (63.63)	34 (35.05)	202 (44.69)	570 (32.00)
No	9 (20.45)	41 (42.26)	112 (24.77)	668 (37.50)
Maybe	5 (11.36)	6 (6.185)	61 (13.49)	227 (12.74)
I don't know	2 (4.54)	16 (16.4)	77 (17.0)	316 (17.7)
**Total (n)**	**44**	**97**	**452**	**1781**
**Importance of mentorship**				
Very important	28 (63.63)	38 (39.17)	239 (52.87)	582 (32.67)
Somewhat important	13 (29.54)	27 (27.83)	151 (33.40)	686 (38.51)
Not very important	0 (0)	10 (10.30)	26 (5.75)	159 (8.92)
Not important at all	1 (2.27)	0 (0)	2 (0.44)	17 (0.95)
**Total (n)**	**42**	**75**	**418**	**1444**

## Discussion

The present study examined the characteristics of LGBTQ+ dual degree trainees, factors associated with career interests and goals, and potential barriers experienced by LGBTQ+ physician-scientist trainees. Several previous studies of dual degree trainees have been conducted at a national level [33–38]. However, to our knowledge, no national study to date has included questions about gender identity and sexual orientation. Studies at the institutional level likewise have not examined experiences of LGBTQ+ dual degree trainees, possibly because the low number of LGBTQ+ trainees enrolled at a given time would make these trainees’ information identifiable. Nationally available data on LGBTQ+ dual degree trainees is limited. The Association of American Medical Colleges (AAMC) first included a question on gender identity in their Matriculating Student Questionnaire in 2016 [39], and as part of the American Medical College Application Service (AMCAS) medical school application in 2018[40–42]. As of the 2022 application cycle, the AAMC Electronic Residency Application Service® (ERAS®) has yet to include a question on gender identity [43]. Questions on gender identity and sexual orientation are not routinely collected at any level [44]. The lack of systematic data collection limits our knowledge about the characteristics, needs, and interests of LGBTQ+ physicians, scientists, and especially physician-scientists [20] and perpetuates the sense of invisibility reported by clinicians and researchers [5, 23–29].

### Respondent characteristics

Respondent characteristics generally parallel the demographics of medical trainees. In the present study, we found that LGBTQ+ trainees account for 8.70% of dual degree and 5.20% of medical survey respondents, despite making up 10–15% of the US population [9]. Our findings add to growing evidence that LGBTQ+ trainees are underrepresented [7, 45, 46], and less likely to remain in STEM fields [11].

### Residency and career plans

The prioritization of patient care by LGBTQ+ dual degree trainees may reflect the desire to improve the experience of LGBTQ+ patients in healthcare settings. Many LGBTQ+ patients report bias and discrimination when seeking healthcare [1]. It is likely that survey respondents have personally experienced or witnessed this bias and discrimination in healthcare settings [17]. Beyond a desire for patient care experiences, a greater percentage of dual degree LGBTQ+ trainees expressed interest in education and advocacy. LGBTQ+ trainees may feel a responsibility to advocate for their community and educate peers and other healthcare professionals about the distinct health needs and disparities experienced by the LGBTQ+ community [5]. Similarly, a greater number of LGBTQ+ trainees identified opportunities to teach as an important factor in career selection. Overall, these data suggest that mentors of LGBTQ+ trainees should be attentive to their mentees’ interest in community engagement, and should help trainees find routes to prepare for these pursuits.

### Barriers in career development for LGBTQ+ trainees

All dual degree trainees identified balancing family and work responsibilities and a lack of opportunity or funding as top career obstacles. In the present study, a greater percentage of LGBTQ+ dual degree trainees compared to non-LGBTQ+ dual degree trainees indicated that they had already experienced barriers to their career related to a lack of opportunity or funding. Cech and Waidzunas (2021) found that LGBTQ+ professionals in STEM fields were more likely to experience career limitations and professional devaluation compared to their non-LGTBQ+ peers [19]. More concerning is that 15.50% of LGBTQ+ dual degree trainees identified sexual harassment as a past hindrance to their career advancement. A zero-tolerance policy on sexual harassment means that any sexual harassment is too much, but it is notable that LGBTQ+ dual degree trainees had such a high prevalence of having experienced sexual harassment. Training program leadership should take into consideration that trainees’ designation of sexual harassment as a “past hindrance” may refer to trainees’ years in their medical or dual degree programs, and as our survey did not inquire into current barriers, we cannot rule out that the harassment is not ongoing. These findings indicate important areas for future investigations.

Discrimination and bias were identified as past barriers more frequently by all LGBTQ+ trainees compared to all non-LGBTQ+ trainees, aligning with results from a recent study found that one in three LGBTQ+ adults in the United States have faced some form of discrimination in the past year [47]. Studies have also shown greater discrimination and harassment of LGBTQ+ people in STEM [19, 45], medical school [48], and residency training [49]. Mansh and colleagues (2015) found that 29.5% of LGBTQ+ trainees surveyed concealed their identity in medical school, with 43.5% citing fear of bias and harassment as a reason for concealing their sexual identity [50]. A recent (2019) systematic review of LGBTQ+ bias reduction programs for healthcare students [51] found that bias-focused educational interventions were effective at increasing knowledge of LGBTQ+ healthcare, experiential learning interventions increased comfort levels working with LGBTQ+ patients, and intergroup contact promoted positive or more tolerant attitudes toward LGBTQ+ patients. Given our findings of the frequency at which LGBTQ+ dual degree trainees have experienced discrimination or harassment, it is vitally important that these types of interventions be adopted broadly at schools and programs across the nation.

### Mentoring can support LGBTQ + trainees

Mentoring relationships are one way trainees can get support in navigating harassment and bias in institutions and are key resources for early career researchers. Mentorship is likely of even greater importance for LGBTQ+ trainees who will face additional barriers related to bias and discrimination compared to their peers, and has been shown to increase the retention of LGBTQ+ trainees in STEM fields [11]. In the present study, substantially more LGBTQ+ trainees indicated they believe their mentors advocate for them compared to non-LGBTQ+ dual degree trainees, which may suggest strong mentoring relationships and/or more instances in which a mentor’s advocacy is needed for these trainees. The majority of LGBTQ+ trainees also identified mentorship as being important to their careers. LGBTQ+ trainees may struggle to find mentors who identify as LGBTQ+ since LGBTQ+ status is not routinely recognized in medicine. As a result, LGBTQ+ students, residents, and faculty are not identified in a consistent way across institutions. LGBTQ+ trainees seek out potential mentors through institutional websites, interest groups, or other resources on campus, which vary between programs and may be difficult to navigate [52]. Another limitation is that resources and mentoring opportunities are often dependent on the leadership and participation of a limited number of LGBTQ+ faculty at each institution, and interest groups and resources for LGBTQ+ trainees may dissolve if a key faculty member transitions to a new institution or position. Despite these potential barriers, LGBTQ+ trainees clearly benefit from the advocacy and support of mentors. Programs should intentionally support communities for LGBTQ+ trainees and help students identify mentorship opportunities. Schools should also leverage the power of online connections by assisting trainees in finding LGBTQ+ mentors in their fields through existing online mechanisms and communities [53].

### Limitations and future directions

This study has several limitations. Our study sample size was small, mirroring the underrepresentation of LGBTQ+ individuals in medicine [10]. However, despite the small sample size, our work still represents the largest study of LGBTQ+ dual degree trainees conducted to date. This observational study cannot draw conclusions about the influence of factors we observed regarding LGBTQ+ trainees’ experiences or decisions in training. Another important limitation is that we did not include questions on the unique experiences of transgender and non-binary trainees who are often further marginalized in academic medical culture [23]. Future studies should explicitly include questions about the experiences of transgender and non-binary trainees to avoid further exclusion, and explore the motivation behind question responses. Finally, our survey ended in 2014, meaning that this data is six years old. It will be important to conduct a similar survey soon given the tremendous work in this area by several national organizations, including the National Institutes of Health, to discern whether changes have occurred during this time.

## Conclusions

This is the first and largest study to characterize LGBTQ+ MD/PhD and DO/PhD trainees. LGBTQ+ physician scientist trainees remain under-represented and under-studied, especially women, transgender, and non-binary trainees. LGBTQ+ physician scientist trainees face unique barriers during their training as well as higher rates of discrimination and harassment. Despite a desire for visibility, the unique needs and values of LGBTQ+ trainees will remain under-recognized until data about sexual orientation and gender identity are routinely and systematically captured.

## Supplementary Information

**Additional file 1.**

## Data Availability

Due to IRB stipulation, the data will not be publicly available, but can be requested from corresponding author, Dr. Kwan.

## Abbreviations

LGBTQ+: Lesbian, gay, bisexual, transgender, queer, questioning, non-binary, non-conforming, genderqueer, intersex, asexual, two-spirit, and/or non-cisgender
STEM: Science, technology, engineering and mathematics
AAMC: Association of American Medical Colleges
AMCAS: American Medical College Application Service
ERAS: Electronic Residency Application Service®

## References

[1] Rossman K, Salamanca P, Macapagal K (2017). A qualitative study examining young adults’ experiences of disclosure and nondisclosure of LGBTQ identity to health care providers. J Homosex.

[2] Martos AJ, Wilson PA, Gordon AR, Lightfoot M, Meyer IH (2018). “Like finding a unicorn”: healthcare preferences among lesbian, gay, and bisexual people in the United States. Soc Sci Med.

[3] Rutherford K, Mcintyre J, Daley A, Ross LE (2012). Development of expertise in mental health service provision for lesbian, gay, bisexual and transgender communities. Med Educ.

[4] Gonzales G, Przedworski J, Henning-Smith C (2016). Comparison of health and health risk factors between lesbian, gay, and bisexual adults and heterosexual adults in the United States: results from the national health interview survey. JAMA Intern Med.

[5] Powell K, Terry R, Chen S (2020). How LGBT+ scientists would like to be included and welcomed in STEM workplaces. Nature.

[6] Cooper KM, Auerbach AJJ, Bader JD, Beadles-Bohling AS, Brashears JA, Cline E (2020). Fourteen recommendations to create a more inclusive environment for lgbtq+ individuals in academic biology. CBE Life Sci Educ.

[7] Freeman J (2018). LGBTQ scientists are still left out comments. Nature.

[8] Phelan SM, Burke SE, Hardeman RR, White RO, Przedworski J, Dovidio JF (2017). Medical school factors associated with changes in implicit and explicit bias against gay and lesbian people among 3492 graduating medical students. J Gen Intern Med.

[9] GLAAD. Accelerating Acceptance 2017. 2017. Available from: https://www.glaad.org/files/aa/2017_GLAAD_Accelerating_Acceptance.pdf

[10] Tanner L. U.S. medical schools boost LGBTQ students, doctor training - ABC News. Associated Press. 2020 Feb 20 [cited 30 May 2021]; Available from: https://abcnews.go.com/Health/wireStory/us-medical-schools-boost-lgbtq-students-doctor-training-69073544

[11] Hughes BE. Coming out in STEM: Factors affecting retention of sexual minority STEM students. Sci Adv. 2018;4(3). 10.1126/sciadv.aao6373 PMC5851677 29546240

[12] Samuels EA, Boatright DH, Wong AH, Cramer LD, Desai MM, Solotke MT (2021). Association between sexual orientation, mistreatment, and burnout among US medical students. JAMA Netw Open.

[13] Nadal KL (2019). Queering and browning the pipeline for LGBTQ faculty of color in the academy: the formation of the LGBTQ scholars of color national network. J Crit Thought Prax.

[14] University of Colorado Boulder. CU Boulder 2014 Graduate Student Social Climate Survey: Report on Differences by Gender, Sexual Orientation and Race/Ethnicity. 2017 [cited 30 May 2021]. Available from: https://www.colorado.edu/oda/sites/default/files/attached-files/2014graddemoanalysis_0.pdf

[15] Mancini O (2011). Attrition risk and resilience among sexual minority college students. Columbia Soc Work Rev.

[16] Kosciw JG, Clark CM, Truong NL, Zongrone AD. The 2019 National School Climate Survey: The Experiences of Lesbian, Gay, Bisexual, Transgender, and Queer Youth in Our Nation’s Schools. A Report from GLSEN. Gay, Lesbian Straight Educ Netw. 2020; Available from: www.glsen.org/research.

[17] de Bourmont SS, Burra A, Nouri SS, El-Farra N, Mohottige D, Sloan C (2020). Resident physician experiences with and responses to biased patients. JAMA Netw Open.

[18] Dyer J, Townsend A, Kanani S, Matthews P, Palermo A. Exploring the workplace for LGBT+ physical scientists. R Soc Chem Inst Physics, R Astron Soc. 2019. Available from: https://www.rsc.org/globalassets/04-campaigning-outreach/campaigning/lgbt-report/lgbt-report_web.pdf.

[19] Cech EA, Waidzunas TJ (2021). Systemic inequalities for LGBTQ professionals in STEM. Sci Adv.

[20] Langin K (2021). LGBTQ researchers say they want to be counted. Science.

[21] Association of American Medical Colleges. Underrepresented in Medicine Definition - Initiatives - AAMC. 2018 [cited 30 May 2021]. Available from: https://www.aamc.org/what-we-do/equity-diversity-inclusion/underrepresented-in-medicine

[22] National Institute of Health. Populations Underrepresented in the Extramural Scientific Workforce. 2020 [cited 30 May 2021]. Available from: https://diversity.nih.gov/about-us/population-underrepresented

[23] Cook TE, Dimant OE, Novick R, Adegbola A, Blackstock U, Drake CB (2020). Gendered expectations: strategies for navigating structural challenges in support of transgender and nonbinary trainees in academic medicine. Acad Med.

[24] Mattheis A, De Arellano DCR, Yoder JB (2020). A model of queer STEM identity in the workplace. J Homosex.

[25] Bilimoria D, Stewart AJ. “Don’t ask, don’t tell”: The academic climate for lesbian, gay, bisexual, and transgender faculty in science and engineering. NWSA J. 2009;21(2):85-103.

[26] Cech EA, Waidzunas TJ. Navigating the heteronormativity of engineering: The experiences of lesbian, gay, and bisexual students. Eng Stud. 2011;3(1):1-24.

[27] Miller RA, Vaccaro A, Kimball EW, Forester R. “It’s Dude Culture”: Students With Minoritized Identities of Sexuality and/or Gender Navigating STEM Majors. J Divers High Educ. 2020;14(3):340–52. Available from: https://www.science.org/content/article/how-many-scientists-are-lgbtq-federal-survey-delays-frustrate-researchers.

[28] Yoder JB, Mattheis A (2016). Queer in STEM: workplace experiences reported in a national survey of LGBTQA individuals in science, technology, engineering, and mathematics careers. J Homosex.

[29] Langin K. How many scientists are LGBTQ? Federal survey delays frustrate researchers. Science (80- ). 2020.

[30] Kwan JM, Daye D, Schmidt M Lou, Conlon CM, Kim H, Gaonkar B (2017). Exploring intentions of physician-scientist trainees: factors influencing MD and MD/PhD interest in research careers. BMC Med Educ.

[31] Kwan JM, Toubat O, Harrison AM, Riddle M, Wu B, Kim H (2020). A nationwide assessment of perceptions of research-intense academic careers among predoctoral MD and MD-PhD trainees. J Clin Transl Sci.

[32] IBM Corp (2017). IBM SPSS Statistics for Windows.

[33] Andriole DA, Jeffe DB, Hageman HL, Ephgrave K, Lypson ML, Mavis B (2010). Variables associated with full-time faculty appointment among contemporary U.S. Medical school graduates: implications for academic medicine workforce diversity. Acad Med.

[34] Jeffe DB, Andriole DA (2011). A national cohort study of MD-PhD graduates of medical schools with and without funding from the national institute of general medical sciences’ medical scientist training program. Acad Med.

[35] Andriole DA, Whelan AJ, Jeffe DB (2008). Characteristics and career intentions of the emerging MD/PhD workforce. JAMA.

[36] Andriole DA, Jeffe DB (2016). Predictors of full-time faculty appointment among MD-PhD program graduates: a national cohort study. Med Educ Online.

[37] Kosik RO, Tran DT, Fan APC, Mandell GA, Tarng DC, Hsu HS (2016). Physician scientist training in the United States: a survey of the current literature. Eval Health Prof.

[38] Nguyen M, Mason HRC, Barrie U, Jeffe DB, Cavazos JE, Ata A (2021). Association between socioeconomic background and MD-PhD program matriculation. J Gen Intern Med.

[39] Association of American Medical Colleges. Matriculating Student Questionnaire Matriculating Student Questionnaire All Schools Summary Report & Individual School Report Executive Summary Background. 2016 [cited 30 May 2021]. Available from: www.aamc.org/data/msq

[40] Tam A (2017). Medical School Admissions: An LGBTQ Perspective. Acad Med.

[41] Association of American Medical Colleges (2020). 2021 AMCAS ® Applicant Guide.

[42] Gamble RM, Pregnall AM, Deng A, Ehrenfeld JM, Talley JUS (2021). Medical school admissions and enrollment practices: Status of LGBTQ inclusivity. J Osteopath Med.

[43] ERAS 2022-MyERAS Residency User Guide. 2021.

[44] Streed CG, McCarthy EP, Haas JS (2017). Association between gender minority status and self-reported physical and mental health in the United States. JAMA Int Med.

[45] Freeman JB. Measuring and Resolving LGBTQ Disparities in STEM. Policy Insights from Behav Brain Sci. 2020;7(2):141-8.

[46] Sansone D, Carpenter CS. Turing’s children: Representation of sexual minorities in STEM. PLoS One. 2020;15(11 November):e0241596. 10.1371/journal.pone.0241596 PMC7673532 33206668

[47] Gruberg S, Mahowald L, Halpin J. The State of the LGBTQ Community in 2020 - Center for American Progress. 2020 [cited 30 May 2021]. Available from: https://www.americanprogress.org/issues/lgbtq-rights/reports/2020/10/06/491052/state-lgbtq-community-2020/

[48] Hill KA, Samuels EA, Gross CP, Desai MM, SitkinZelin N, Latimore D (2020). Assessment of the prevalence of medical student mistreatment by sex, race/ethnicity, and sexual orientation. JAMA Intern Med.

[49] Pololi LH, Brennan RT, Civian JT, Shea S, Brennan-Wydra E, Evans AT (2020). Us, Too. Sexual harassment within academic medicine in the United States. Am J Med.

[50] Mansh M, White W, Gee-Tong L, Lunn MR, Obedin-Maliver J, Stewart L (2015). Sexual and gender minority identity disclosure during undergraduate medical education: “in the closet” in medical school. Acad Med.

[51] Morris M, Cooper RL, Ramesh A, Tabatabai M, Arcury TA, Shinn M (2019). Training to reduce LGBTQ-related bias among medical, nursing, and dental students and providers: a systematic review. BMC Med Educ.

[52] Jann JT, Edmiston EK, Ehrenfeld JM (2015). Important considerations for addressing LGBT health care competency. Am J Public Health.

[53] 500 Queer Scientists Visibility Campaign : 500 Queer Scientists. [cited 30 May 2021]. Available from: https://500queerscientists.com/

